# Codon modification of *Tuba1a* alters mRNA levels and causes a severe neurodevelopmental phenotype in mice

**DOI:** 10.1038/s41598-023-27782-2

**Published:** 2023-01-21

**Authors:** Ines Leca, Alexander William Phillips, Lyubov Ushakova, Thomas David Cushion, David Anthony Keays

**Affiliations:** 1 Vienna Biocenter (VBC), Research Institute of Molecular Pathology (IMP), Campus-Vienna-Biocenter 1, 1030 Vienna, Austria; 2 Department of Physiology, Development and Neuroscience, University of Cambridge, Downing Street, Cambridge, CB2 3EG UK; 3 Department of Biology, Ludwig-Maximilians-University Munich, 82152 Planegg-Martinsried, Germany

**Keywords:** Molecular biology, Neuroscience, Development of the nervous system, Diseases of the nervous system, Genetics of the nervous system

## Abstract

The tubulinopathies are an umbrella of rare diseases that result from mutations in tubulin genes and are frequently characterised by severe brain malformations. The characteristics of a given disease reflect the expression pattern of the transcript, the function of a given tubulin gene, and the role microtubules play in a particular cell type. Mouse models have proved to be valuable tools that have provided insight into the molecular and cellular mechanisms that underlie the disease state. In this manuscript we compare two *Tuba1a* mouse models, both of which express wild-type TUBA1A protein but employ different codon usage. We show that modification of the *Tuba1a* mRNA sequence results in homozygous lethality and a severe neurodevelopmental phenotype. This is associated with a decrease in the number of post-mitotic neurons, PAX6 positive progenitors, and an increase in the number of apoptotic cells. We attribute this to a decrease in the stability of the modified *Tuba1a* transcript, and the absence of compensation by the other neurogenic tubulins. Our findings highlight the importance of maintaining the wild-type coding sequence when engineering mouse lines and the impact of synonymous genetic variation.

## Introduction

Microtubules are long and hollow cylinders that participate in many fundamental cellular functions^1^. During mitosis, they make up the mitotic spindle, they act as a scaffold for intracellular trafficking and, because they are a component of cilia and flagella, they contribute to cell motility. To render such a diversity of mechanical tasks, microtubules undergo cycles of polymerisation and de-polymerisation, a stochastic behaviour termed ‘dynamic instability’^2^. They are assembled from α- and β-tubulin heterodimers that fold via a highly conserved and complex pathway involving chaperones, chaperonins and other co-factors^3^. One feature of microtubules is that α- and β-tubulins derive from a multi-gene family, which share a high degree of sequence homology with each other and with their orthologs. Nonetheless, the tubulin isoforms are distinctly different. They are encoded by genes on different chromosomes, their carboxy-terminal and 3’ untranslated-region are varied, and they have distinct and interesting expression patterns. To date much research has focused on the α-tubulin *TUBA1A* as it is highly expressed in the developing nervous system and de novo mutations cause a broad spectrum of diseases, including lissencephaly and microcephaly^4–9^. To gain insight into the underlying molecular pathology and function of TUBA1A investigators have exploited both spontaneous and mutant mouse models^5,10,11^. We have previously shown that a S140G mutation in mice causes defects in neuronal migration, and Stottmman and colleagues have reported that ablation of *Tuba1a* causes perinatal lethality in homozygous mice with severe brain malformations at E16.5^11^. The generation of various reporter lines indicates that TUBA1A is highly expressed in post-mitotic neurons and is largely absent from progenitors^12–14^.

In addition to enhancer driven gene expression tubulin mRNA levels are controlled by an autoregulatory mechanism^15,16^. It has been shown that tubulin protein monomers influence the levels of tubulin mRNA, which is dependent on the first four amino acids of the protein (MREI motif)^15–17^. Lin and colleagues have recently demonstrated that this mechanism relies on the ribosome associated protein TTC5, which recognizes the MREI motif at the N-terminus of the tubulins and triggers degradation of tubulin mRNA^18^. Collectively these studies have highlighted the importance of tubulin mRNA stability, which in turn influences the cytoskeletal ecosystem of the cell. Here, we report the generation of a mouse line that carries an alternative codon sequence in exon 4 of *Tuba1a*^19^. We show that homozygotes expressing the modified *Tuba1a* mRNA present with a severe neurodevelopmental phenotype which is associated with a significant decrease in *Tuba1a* mRNA levels.

## Results

### Generation of two *R402H Tuba1a* conditional mouse lines

**Figure 1:**
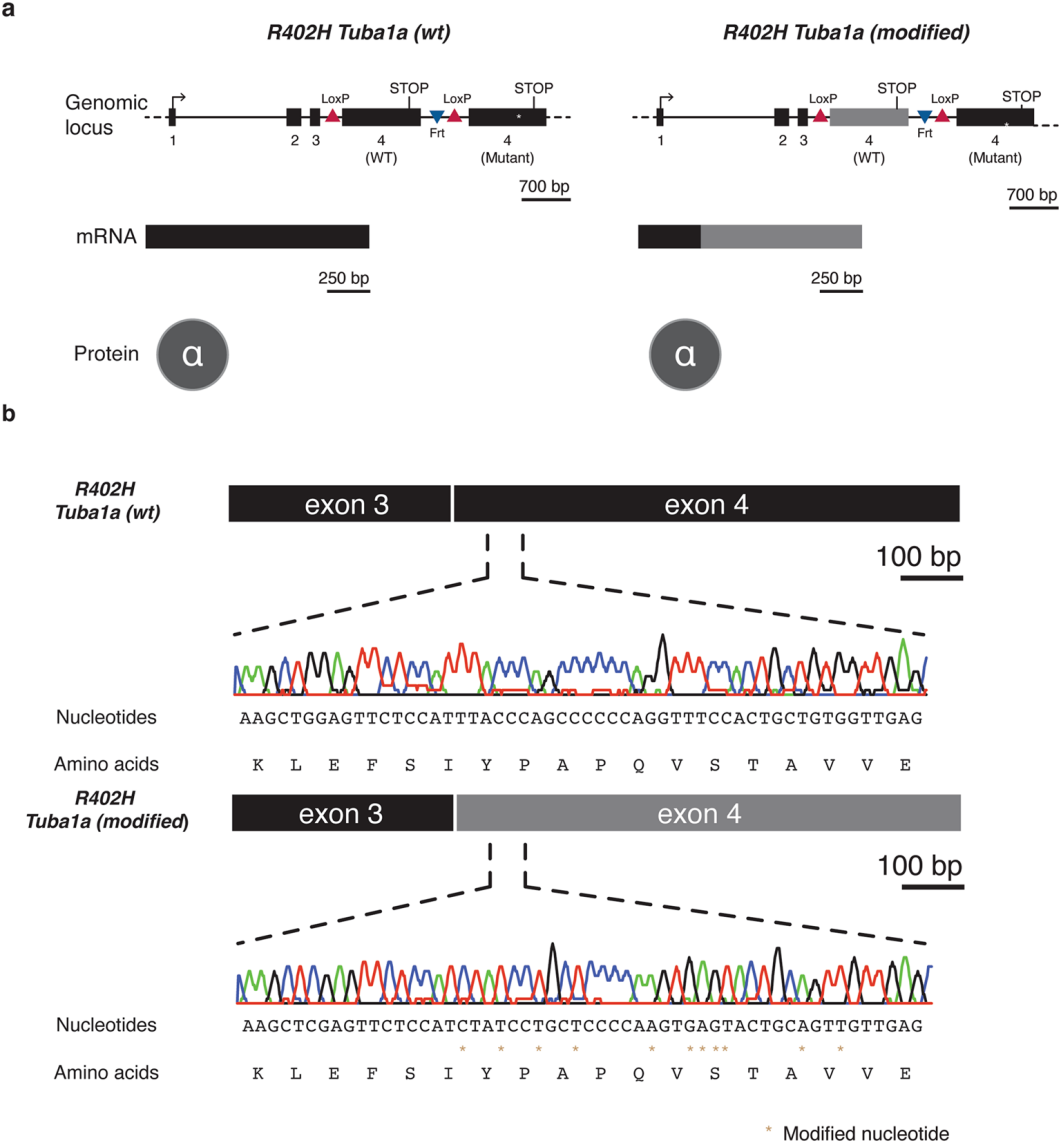
Two *R402H Tuba1a* mouse lines expressing either wild-type or a modified version of exon 4. (**a**) Schematic representation of the genomic locus in both *R402H Tuba1a* conditional mouse lines. In both cases, exon 4 was flanked by LoxP sites to enable the expression of *R402H* mutant *Tuba1a*, in the presence of Cre recombinase. In the *R402H Tuba1a (modified)* line, the codon sequence was altered (in grey). This modification results in a different mRNA sequence compared to the *R402H Tuba1a (wt)* line however, on the protein level, both TUBA1A molecules share the same sequence. (**b**) Sequencing traces from the *R402H Tuba1a (wt)* and *R402H Tuba1a (modified)* lines show the synonymous variations introduced (gold stars).

With the aim of studying the effects of a recurrent patient mutation (R402H) in TUBA1A, we designed a mouse line that permits the conditional expression of the R402H mutation^19^ (Fig. 1a, Supplementary Fig. 1). This line includes two copies of exon 4, with the wild-type sequence flanked by LoxP sites, followed by a second allele with the R402H mutation. In the first instance we changed the codon sequence of the WT allele, to limit promiscuous homologous recombination when targeting in ES cells. This line we will refer to as R402H *Tuba1a* (modified). We then created a second line that was identical in all respects, except the codon sequence was not modified, which we refer to as R402H *Tuba1a (wt)*. For this study neither line was crossed with a Cre driver, and therefore the R402H mutation is not expressed^19^. We simply compare two lines that both express the wild-type TUBA1A protein, but one with modified codon usage. To confirm that the R402H mutation is not expressed we extracted mRNA from E16.5 embryos, and generated cDNA. Sanger sequencing showed that the R402H variant is not present in the mRNA pool and that the codon sequence was only altered in exon 4 in *R402H Tuba1a (modified)* mice (Fig. 1b). A sequence comparison revealed that the overall homology between the modified and the wild-type *Tuba1a* allele was 78.8%.

### Perinatal lethality and impaired neurodevelopment in *Tuba1a R402H*^*mod*^*/R402H*^*mod*^ homozygotes

**Figure 2:**
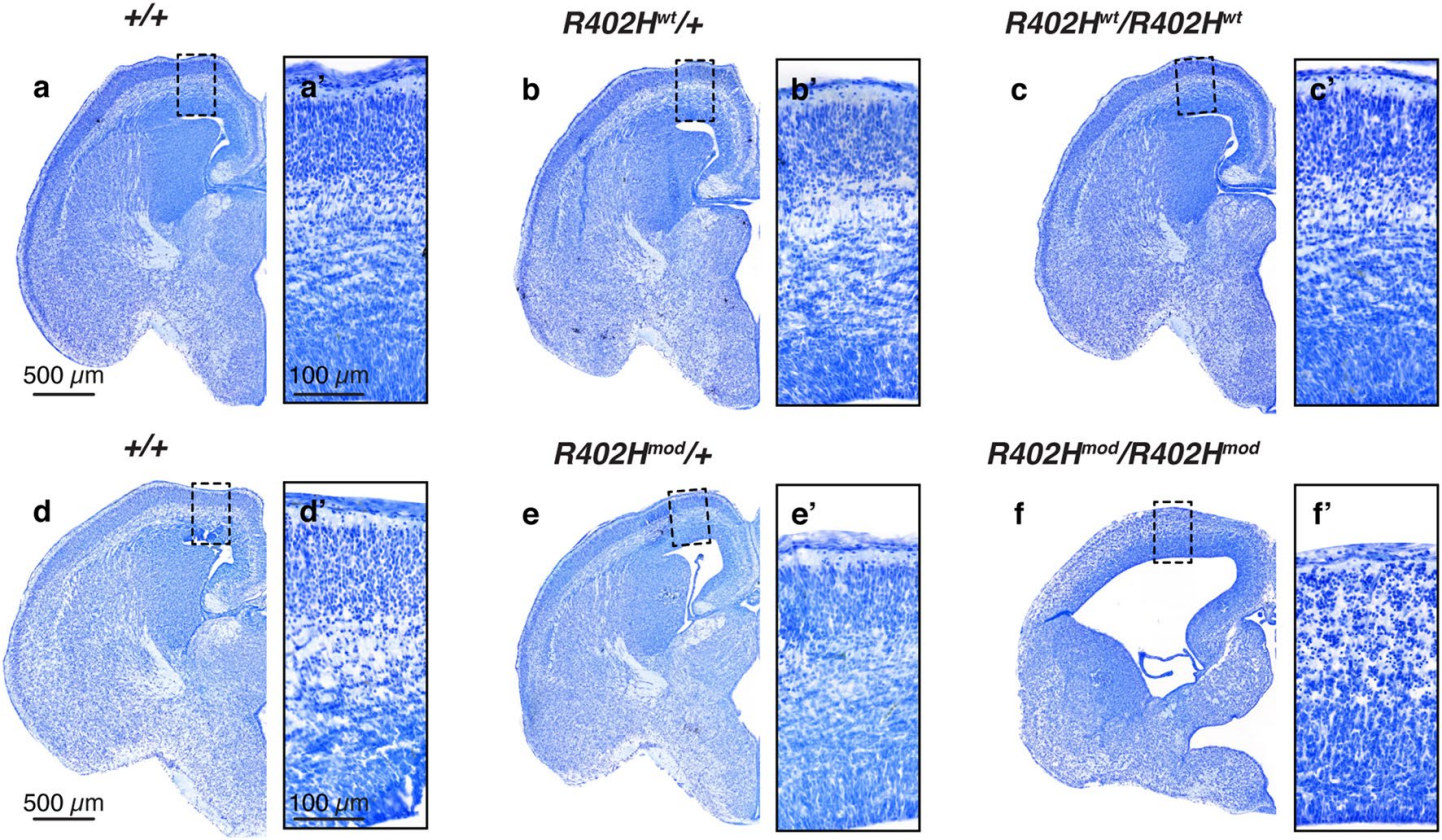
*R402H*^*mod*^*/R402H*^*mod*^ embryos show a severe neurodevelopmental phenotype which is not observed in *R402H*^*wt*^*/R402H*^*wt*^ mice. (**a–c**) Representative coronal sections of E16.5 embryos stained with Nissl. Wild-type (+/+), heterozygous (*R402H*^*wt*^*/*+) and homozygous (*R402H*^*wt*^*/R402H*^*wt*^) animals are indistinguishable. (**a′–c′**) Enlargements of the boxed areas marked in (**a–c**) showing no differences in cortical organization between genotypes. (**d–f**) Representative coronal sections of E16.5 embryos stained with Nissl of wild-type (+/+), heterozygous (*R402H*^*mod*^*/*+) and homozygous (*R402H*^*mod*^*/R402H*^*mod*^) animals. Brain development is significantly impaired in homozygotes, leading to perinatal lethality. (**d′–f′**) Enlargements of the boxed areas marked in (**d–f**) showing a severe cortical disorganization in *R402H*^*mod*^*/R402H*^*mod*^ embryos compared to littermates. Scale bars indicate 500 μm in (**a**) and (**d**), and 100 μm in (**a′**) and (**d′**).

We proceeded by analysing the phenotype of both *R402H Tuba1a (wt)* and *R402H Tuba1a (modified)* lines in the absence of Cre. To our surprise homozygous *R402H*^*mod*^*/R402H*^*mod*^ mice did not survive until birth, so we collected E16.5 embryos from both lines (Fig. 2a–f, Supplementary Fig. 2). *R402H*^*wt*^*/R402H*^*wt*^ animals are completely normal in all respects, are indistinguishable from controls (+/+), and survive until adulthood (Fig. 2a–c, Supplementary Fig. 2a–c)^19^. In contrast, *R402H*^*mod*^*/R402H*^*mod*^ animals present with a severe neurodevelopmental phenotype (Fig. 2d-f–, Supplementary Fig. 2d–f). In comparison to wild-type controls and heterozygotes, these animals show an enlargement of the ventricles, cortical thinning and disorganization, and a reduction in the size of the striatum and thalamus (Fig. 2d–f).

### Reduction in Ctip2 positive neurons and PAX6 positive progenitors in *Tuba1a R402H*^*mod*^*/R402H*^*mod*^ homozygotes

**Figure 3:**
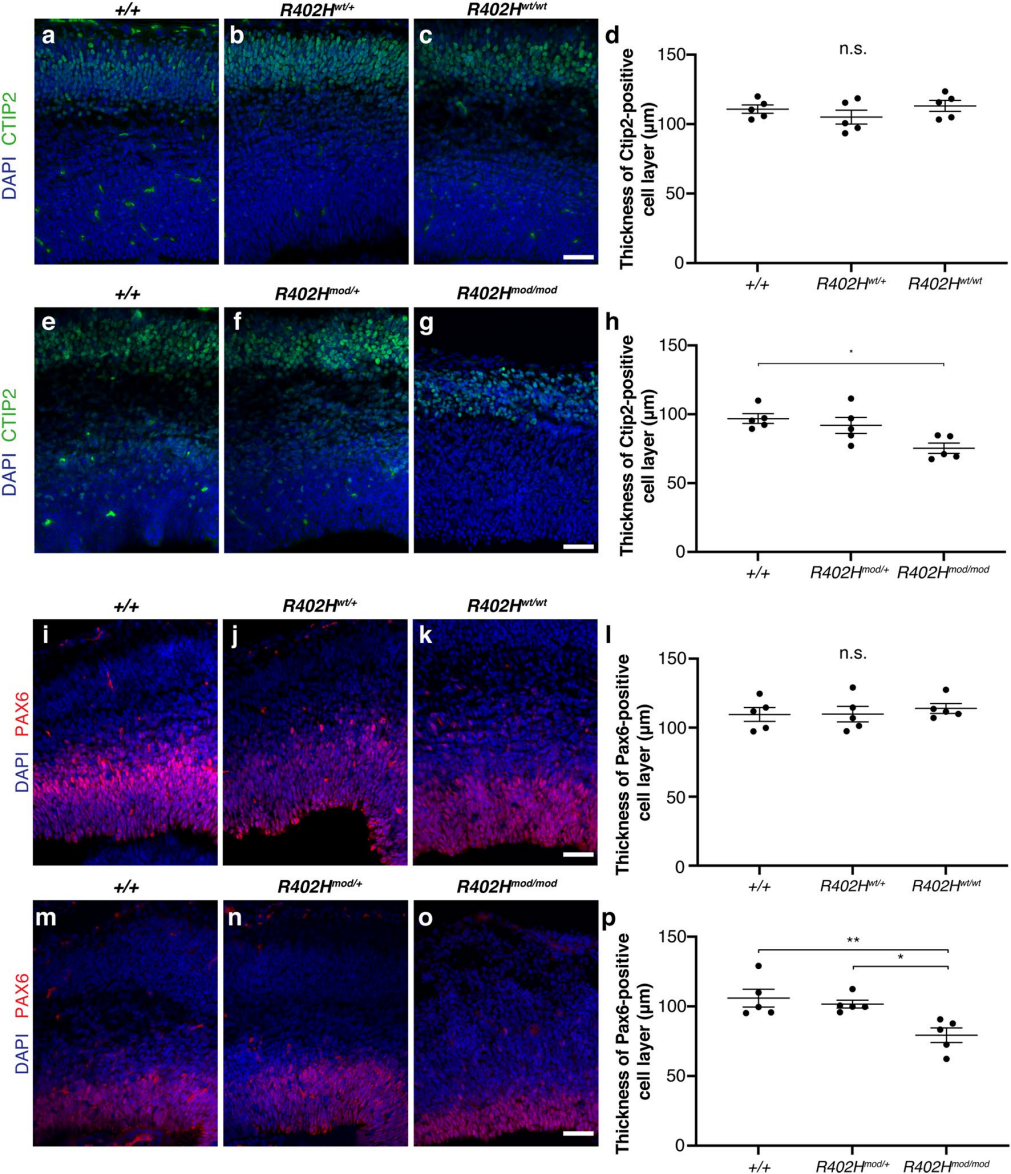
*R402H*^*mod*^*/R402H*^*mod*^ embryos have a reduction in Ctip2 positive neurons and PAX6 positive progenitors. (**a–c**) Coronal sections of E16.5 embryos stained for the layer 5/6 neuronal marker Ctip2 from *Tuba1a* WT animals. (**a**) Wild-type (+/+); (**b**) heterozygous (*R402H*^*wt*^*/*+), and (**c**) homozygous (*R402H*^*wt*^*/R402H*^*wt*^) animals are indistinguishable. (**d**) Quantification of Ctip2-positive neuronal layer reveals no significant different between homozygous animals and control littermates. (**e–g**) Representative coronal sections from *Tuba1a* modified embryos stained for Ctip2. (**e**) wild-type (+/+); (**f**) heterozygous (*R402H*^*mod*^*/*+) and (**g**) homozygous (*R402H*^*mod*^*/R402H*^*mod*^) animals. (**h**) Quantification shows a significant reduction in Ctip2 layer thickness in homozygous *R402H*^*mod*^*/R402H*^*mod*^ compared to wildtype littermates. Scale bars indicate 50 μm. (**i–k**) Representative coronal sections from E16.5 embryos stained for neural progenitor marker Pax6. (I) Wild-type (+ / +); (**j**) heterozygous (*R402H*^*wt*^*/*+), and (**k**) homozygous (*R402H*^*wt*^*/R402H*^*wt*^) animals. (**l**) Quantification of ventricular zone thickness shows no significant different between homozygous animals and control littermates. (**m–o**) Coronal sections from *Tuba1a* modified embryos stained for Pax6. (**m**) wild-type (+/+); (**n**) heterozygous (*R402H*^*mod*^*/*+) and (**o**) homozygous (*R402H*^*mod*^*/R402H*^*mod*^) animals. (**p**) Quantification shows a significant reduction in VZ thickness in homozygous *R402H*^*mod*^*/R402H*^*mod*^ compared to wildtype littermates. One-way ANOVA with Tukey’s test for multiple comparisons. P* < 0.05, P** < 0.01. Error bars show Mean ± SEM. Scale bars indicate 50 μm.

To further assess the defects in brain morphology in the *R402H*^*mod*^*/R402H*^*mod*^ animals**,** we stained with the neuronal marker Ctip2 which labels layer 5/6 neurons, the dominant neuronal population present at E16.5. Quantitation of layer thickness revealed no significant difference between homozygous R402H^wt/wt^ and littermate controls (n = 5; + */* + vs. *R402H*^*wt*^*/*+ *,* *P* > 0.05; +*/*+ vs. *R402H*^*wt*^*/R402H*^*wt*^, *P* > 0.05) (Fig. 3a–d). In contrast, there was significant reduction in the thickness of the Ctip2-positive neuronal cell layer when comparing homozygous *R402H*^*mod*^*/R402*^*mod*^ animals and littermate controls (n = 5 + / + vs. *R402H*^*mod/mod*^ *p* < 0.05) (Fig. 3e–h). To determine whether the neuroanatomical defects observed in homozygous *R402H*^*mod*^*/R402H*^*mod*^ also affect neural progenitor cells, we stained for the progenitor marker Pax6 and quantified the thickness of the ventricular zone at E16.5. We observed no significant difference in VZ thickness between R402H^wt/wt^ and control littermates (n = 5; +*/*+ vs. *R402H*^*wt*^*/*+ *,* *P* > 0.05; +*/*+ vs. *R402H*^*wt*^*/R402H*^*wt*^, *P* > 0.05) (Fig. 3i–l). In comparison, the thickness of the VZ is significantly reduced in homozygous *R402*^*mod*^*/R402H*^*mod*^ animals compared to heterozygous *R402*^*mod*^*/R402H*^+^ and wild-type control littermates (n = 5; +*/*+ vs. *R402H*^*mod*^*/*^*mod*^*,* *P* < 0.01; *R402H*^*mod*^*/R402H*^+^ vs. *R402H*^*mod*^*/*^*mod*^, *P* < 0.05) (Fig. 3m–p). These data show that modification of the *Tuba1a* locus perturbs the architecture of the ventricular zone as well as altering neuronal cell layers.

### Increase in apoptotic cells in *Tuba1a R402H*^*mod*^*/R402H*^*mod*^ homozygotes

**Figure 4:**
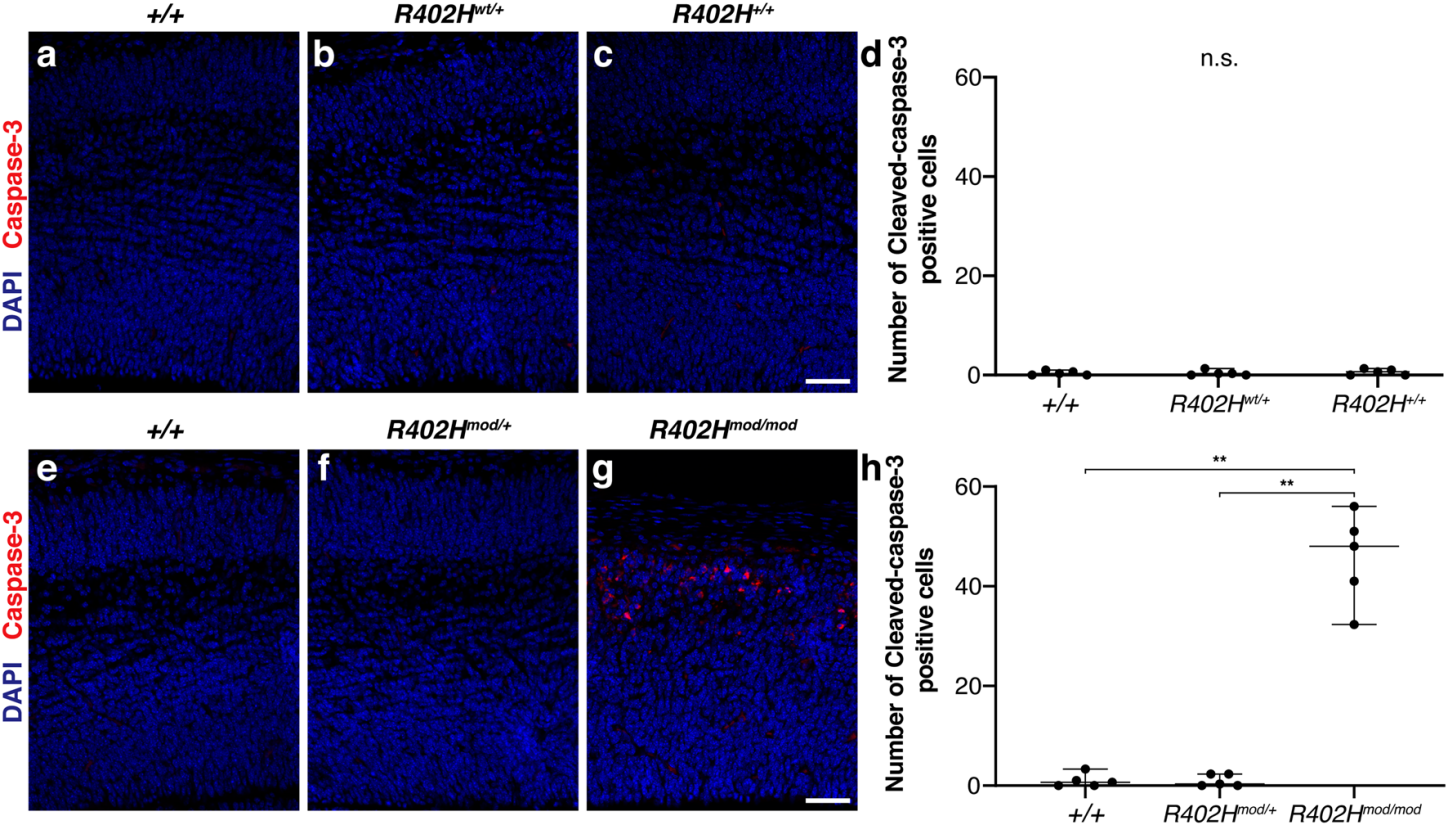
*R402H*^*mod*^*/R402H*^*mod*^ embryos have an increase in the number of apoptotic cells. (**a–c**) Representative coronal sections from E16.5 embryos stained for the apoptotic cell marker cleaved-caspase 3. (**a**) Wild-type (+/+); (**b**) heterozygous (*R402H*^*wt*^*/*+), and (**c**) homozygous (*R402H*^*wt*^*/R402H*^*wt*^) animals contain very few caspase-3-positive cells. (**d**) Quantification of caspase-3-positive cells shows no significant different between homozygous animals and control littermates. (**e–g**) Coronal sections from *Tuba1a* modified embryos stained for Caspase-3. (**e**) wild-type (+/+); (**f**) heterozygous (*R402H*^*mod*^*/*+) and (**g**) homozygous (*R402H*^*mod*^*/R402H*^*mod*^) animals. (**h**) Quantification shows that homozygous *R402H*^*mod*^*/R402H*^*mod*^ have a significant increase in the number of caspase-3-positive cells compared to wildtype and heterozygous *R402H*^*mod*^*/R402H*^+^ littermates. *P* < 0.01**. Error bars show Mean ± SEM. Scale bars indicate 50 μm.

Mutations in *Tuba1a* have previously been associated with an increase in the number of apoptotic cells in mutant animals^11^. To assess whether homozygous *R402H*^*mod*^*/R402H*^*mod*^ mice have increased apoptosis compared to control animals, we performed staining for the apoptotic cell marker cleaved caspase-3 and quantified the number of apoptotic cells in mutant and control mice. Homozygous *R402H*^*wt*^*/R402H*^*wt*^ animals have very few capsase-3-positive cells similar to control littermates (Fig. 4a–d). In comparison, we observed many caspase-3-positive cells in homozygous *R402H*^*mod*^*/R402H*^*mod*^ mice (Fig. 4e–g). Quantification revealed a significant increase in the number of apoptotic cells in *R402H*^*mod*^*/R402H*^*mod*^ compared to heterozygous *R402H*^*mod*^*/R402H*^+^ and wild-type littermates (n = 5; +*/*+ vs. *R402*^*mod*^*/R402*^+^*,* *P* < 0.01; *R402*^*mod*^*/R402*^+^ vs. *R402H*^*mod*^*/R402H*^*mod*^, *P* < 0.01) (Fig. 4h). These results demonstrate that expression of the modified *Tuba1a* allele leads to increased apoptosis during the development of the cortex.

### *Tuba1a *mRNA levels are significantly reduced in homozygous *R402H*^*mod*^*/R402H*^*mod*^ embryos

**Figure 5:**
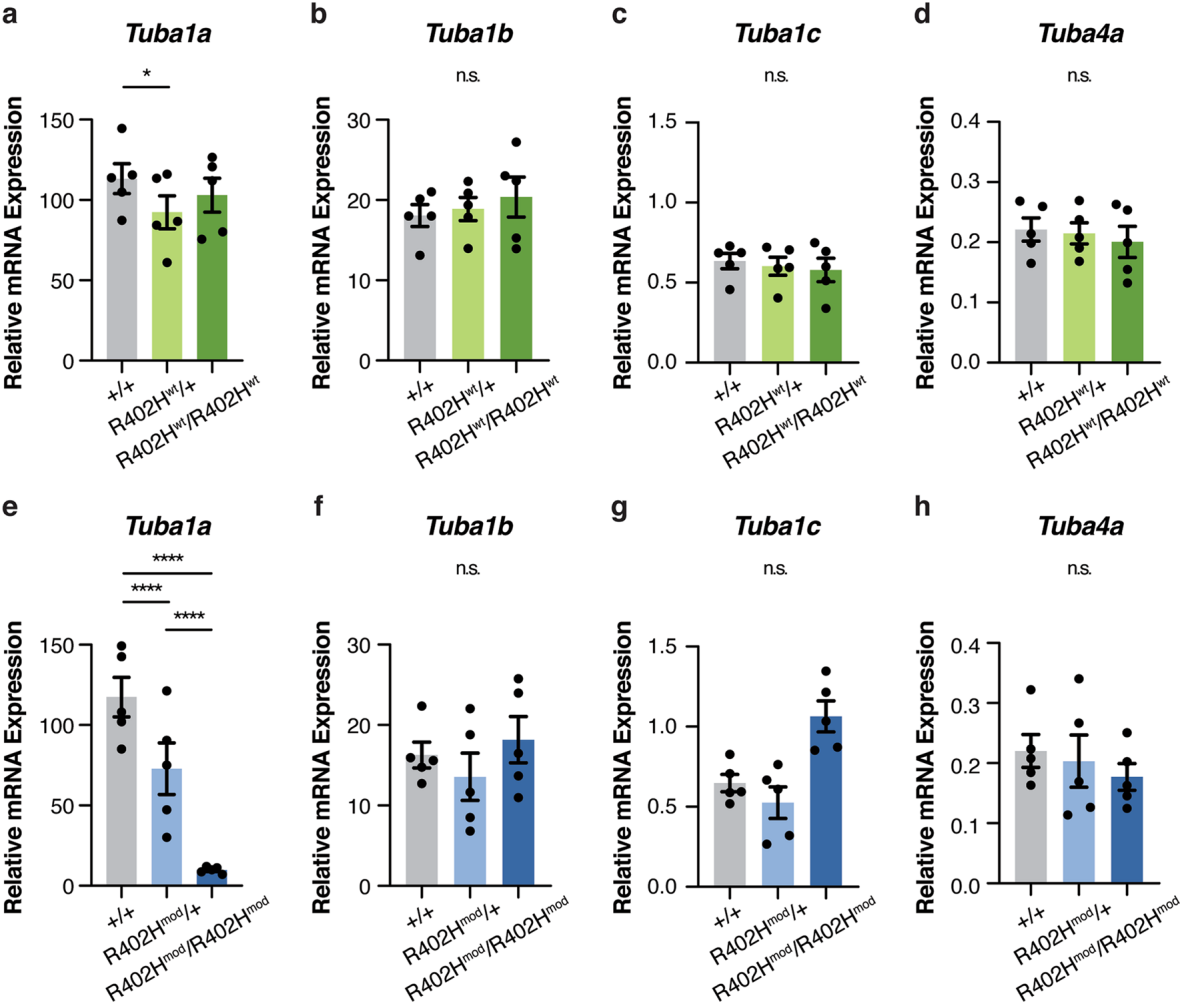
Expression levels of mouse α-tubulin genes in the developing brain are perturbed in the *R402H Tuba1a (modified)* line. (**a–d**) mRNA expression levels of *Tuba1a* (**a**)*, Tuba1b* (**b**)*, Tuba1c* (**c**) and *Tuba4a* (**d**) were assessed by qPCR in E16.5 wild-type (grey), heterozygous (light green), and homozygous *R402H*^*wt*^*/R402H*^*wt*^ (dark green) embryos. We found a slight reduction in the expression levels of *Tuba1a* levels between wild-type and heterozygous animals (n = 5, +*/*+ vs. *R402H*^*wt*^*/*+ *P* < 0.05). (**e–h**) mRNA expression levels of *Tuba1a* (**e**)*, Tuba1b* (**f**)*, Tuba1c* (**g**) and *Tuba4a* (**h**) were assessed by qPCR in E16.5 wild-type (grey), heterozygous (light blue), and homozygous *R402H*^*mod*^*/R402H*^*mod*^ (dark blue) embryos. There was a significant reduction in *Tuba1a* levels in both heterozygous and homozygous animals (n = 5, +*/*+ vs. *R402H*^*mod*^*/*+ *P* < 0.0001; +*/*+ vs. *R402H*^*mod*^*/R402H*^*mod*^ *P* < 0.0001 and *R402H*^*mod*^*/*+ vs. *R402H*^*mod*^*/R402H*^*mod*^ *P* < 0.0001). Error bars show mean ± s.e.m.. Two-way repeated measures ANOVA with Tukey’s test for multiple comparisons; n.s.—not significant; *P < 0.05; ****P < 0.0001.

To gain insight into this phenotype, we assessed the expression levels of the neuronal α-tubulins: *Tuba1a*, *Tuba1b*, *Tuba1c* and *Tuba4a* (Fig. 5)^20^. We isolated the cortices of E16.5 embryos, extracted the RNA and performed qPCR on reverse transcribed cDNA. Using isoform-specific primers, we found that the levels of *Tuba1a* were slightly reduced in heterozygous *R402H*^*wt*^*/*+ embryos compared to wild-type littermates although no differences were observed in homozygotes (n = 5; +*/*+ vs. *R402H*^*wt*^*/*+ *,* *P* < 0.05; +*/*+ vs. *R402H*^*wt*^*/R402H*^*wt*^, *P* > 0.1) (Fig. 5a). We found no significant differences in the expression levels of *Tuba1b*, *Tuba1c*, and *Tuba4a* when comparing +*/*+ *, R402H*^*wt*^*/*+ *,* and *R402H*^*wt*^*/R402H*^*wt*^ littermates (See Supplementary Table S1). In contrast the transcript levels of *Tuba1a* were drastically reduced in both heterozygous and homozygous animals with the modified codon sequence (n = 5, +*/*+ vs. *R402H*^*mod*^*/*+ *P* < 0.0001; +*/*+ vs. *R402H*^*mod*^*/R402H*^*mod*^ *P* < 0.0001 and *R402H*^*mod*^*/*+ vs. *R402H*^*mod*^*/R402H*^*mod*^ *P* < 0.0001) (Fig. 5e). We did observe an increase in the levels of *Tuba1c* in *R402H*^*mod*^*/R402H*^*mod*^ animals, but this increase was not significant (n = 5; +*/*+ vs. *R402H*^*mod*^*/R402H*^*mod*^ *P* > 0.5) (Fig. 5b–d,f–h). Collectively these data show that modifying the mRNA sequence of Tuba1a drastically reduces transcript levels.

## Discussion

In this study we describe a new *Tuba1a* mouse line (*R402H*^*mod*^) which expresses wild-type TUBA1A protein in the absence of Cre recombinase, but employs alternative codons^19^. We compared these animals to another line that was identical in all respects, except the codon sequence was not modified (*R402H*^*wt*^). We found that homozygous (*R402H*^*mod*^*/R402H*^*mod*^) animals do not survive until birth and present with a severe neurodevelopmental phenotype at E16.5. This is associated with a decrease in the thickness of the CTIP2-positive neuronal layer and PAX6-positive progenitor layer, as well as an increase in the number of apoptotic cells. We assessed the mRNA expression levels of different α-tubulin isoforms at E16.5 in the developing cortex. Strikingly, both heterozygous (*R402H*^*mod*^*/R402H*^+^) and homozygous animals (*R402H*^*mod*^*/R402H*^*mod*^) showed a significant reduction in *Tuba1a* mRNA levels when compared to *R402H*^+^*/R402H*^+^ controls. The drastic reduction in mRNA levels in homozygous animals suggests that this codon modified allele acts by a hypomorphic mechanism. Indeed, the phenotype we observe at E16.5 is reminiscent of that described by Bittermann and colleagues who generated two CRISPR-mediated *Tuba1a* mutant lines where the entire gene locus was deleted^11^. Loss of *Tuba1a* in both *Tuba1a*^*d4304/d4304*^ and *Tuba1a*^*d4262/d4262*^ mutants was perinatal lethal and E16.5 embryos presented with major cortical malformations. These findings, and the data presented in this study, suggest that the other α-tubulins expressed in the developing brain (*Tuba1b*, *Tuba1c*, *Tuba4a*) are not able to compensate for the loss of *Tuba1a*.

What might be the underlying cause for the reduction in *Tuba1a* mRNA levels we observed in *R402H*^*mod*^*/R402H*^*mod*^ mice? Although the tubulin autoregulatory mechanism seems to depend on the four-initial amino-acids of α- and β-tubulins, we cannot exclude the possibility that the remainder of the transcript plays a role. The altered codons could impair the binding of other proteins that, like TTC5, influence the levels of tubulin mRNAs^18^. Interestingly, a study in *Drosophila* has also shown that different codon usage can affect protein structure as it affects the rate of co-translation protein folding^21^. Given, however, that tubulin folding is mediated by numerous chaperones (e.g., prefoldin, the cytosolic chaperonin CCT and several chaperone proteins termed TBCA-TBCE) we think this is unlikely to underlie the phenotype we observe^3,22^. One explanation for the reduction in mRNA we observe in the *R402H*^*mod*^*/R402H*^*mod*^ mice is that synonymous variation in exon 4 creates novel splice acceptor sites, resulting in a transcript with a premature stop codon that is subject to nonsense mediated decay^23,24^. Indeed there is growing evidence that synonymous point mutations can modulate gene expression levels, by altering splicing in disease states^25^. The most plausible explanation, however, for the reduction in *Tuba1a* transcript is a change in the stability of the transcript. It is known that the codon sequence of a particular mRNA molecule can affect its stability and consequently its levels^26^. The effect of synonymous genetic variation was recently investigated by Shen and colleagues who analysed the effect of 1,866 synonymous mutations in 21 genes in yeast, assessing their effect on fitness and RNA levels. They found that 75% of synonymous mutations were deleterious, which strongly correlated with the relative expression level of the gene^27^. Highlighting the importance of codon usage in mRNA stability, studies in zebrafish have shown that the use of uncommon synonymous codons plays a critical role in triggering the clearance of maternal mRNA during development, facilitating mRNA degradation^28,29^. Collectively these studies show that synonymous genetic changes can alter the levels of a transcript by influencing its splicing, folding, stability and/or translation rate.

A further observation of interest in the reduction in PAX6 progenitors that we observe in *R402H*^*mod*^*/R402H*^*mod*^ mice. This result is intriguing as TUBA1A is largely considered to be a post-mitotic tubulin^13^. Gloster and colleagues have previously studied the expression of this gene in mice employing a LacZ reporter coupled to the *Tα1-Tuba1a* promoter. They observed transgene expression from E9.5, with high levels of expression in differentiating and migrating neurons at E13.5 in the developing cortex. They did observe a small subpopulation of transgene positive cells in the ventricular zones but argued that this followed terminal mitosis and preceded the onset of migration^13^. Coksaygan and colleagues arrived at a similar conclusion when studying the expression of EYFP coupled to the *Tα1-Tuba1a* promoter^14^. Employing birth date labelling they were not able to detect any dividing progenitors that were EYFP positive in the developing cortex. In contrast, Sawamoto and colleagues concluded that *Tuba1a* is expressed in a limited number of progenitors having crossed the *Tα1-Tuba1a* EYFP line with a Nestin-EGFP driver^30^. Most recently single cell sequencing of the developing human and mouse brain, indicate that a portion of radial glia and intermediate progenitors express *Tuba1a*^31,32^.

In summation we demonstrate that altering the codon sequence of *Tuba1a* in mice causes a severe neurodevelopmental phenotype, which is associated with a decrease in the number of post-mitotic neurons and apical progenitors. Moreover, it highlights the importance of codon usage when engineering transgenic mice.

## Methods

### Ethics statement

All experimental protocols in this manuscript were carried out according to legal requirements and were covered by an approved license (M58/006093/2011/14) from the City of Vienna. All methods were carried out in accordance with relevant guidelines and regulations. All methods are reported in accordance with ARRIVE guidelines.

### Generation of two *R402H Tuba1a* mouse lines

Both lines were generated in collaboration with Ozgene (Perth, Australia), taking advantage of their goGermline technology. The targeting constructs were designed carrying either the wild-type sequence of *Tuba1a* or modified in order to express a distinct codon sequence in exon 4 (BankIt2640849 Tuba1a OP819667, BankIt2642162 Tuba1a OP819668). Following electroporation into C57/BL6 ES cells, the positive clones were screened using Nde I digestion and southern blot analysis. The clones that had successfully incorporated the constructs, were injected into blastocysts and the resulting chimeras were backcrossed to C57/BL6 animals. Animals from both lines were genotyped using PCR amplification with the primers as described^19^. We sequenced the entire *Tuba1a* locus of both mouse lines using genomic DNA samples and two sets of primers to amplify and sequence the locus: mTuba1a_F1/GGATGCAAAGTCTACGGATG and mTuba1a _R1/ATCTCCTTGCCAATGGTGTA, together with mTuba1a_F2/GAAGGGTGAGTGAGCTTGTG and mTuba1a_R2/AAAGCACACATTGCCACATA. All mice were housed at the animal research laboratories of the Institute of Molecular Pathology on a 14:10 h light:dark cycle, with food and water provided ad libitum.

### Histological studies

E16.5 embryos (n = 5 for each line) were decapitated and drop-fixed in a solution of 4% paraformaldehyde (Carl Roth, 0335.3). Following fixation overnight, the heads were dehydrated using 30% sucrose and embedded in Neg-50 Frozen Section Medium (Thermo Fisher Scientific, 6502) before storage at − 80 °C. Littermates were sectioned coronally (12 μm thick sections) using a cryostat. Matching sections were chosen and stained with Nissl. Briefly, the slides were washed in PBS before a 3–4 min incubation with Nissl stain (0.1% cresyl violet acetate; Sigma, C5042). The slides were then dehydrated for 3 min in increasing concentrations of ethanol (30%, 70%, 95% and 100%) and transferred to xylol before coverslipping with DPX mounting media. The stained slides were digitized in a Pannoramic 250 Flash II slide scanner (3DHISTECH), equipped with a 20x/0.8 Plan-Apochromat objective.

### Immunohistochemistry

Prior to staining, brain sections from littermate mice were matched based on morphological landmarks and selected for further analysis. Cryosections allowed to equilibrate to room temperature overnight. Antigen retrieval was performed a distinct subset of antibodies where stipulated. Slides with cryosections were briefly washed 3 times in PBS before being placed in a coplin jar with the antigen retrieval buffer (Vector Laboratories, H-3301-250). Slides were heated in a water bath to 95 °C. Slides were then removed and allowed to cool for room temperature for 45 min. Sections were washed 3 times 10 min before incubation with blocking/permeabilisation solution for 1 h at room temperature. Following this, the permeabilization solution was removed and sections were incubated with the primary antibody diluted in blocking solution at 4 °C overnight. Primary antibodies were used at the following dilutions: Ctip2 (Abcam, ab18465, 1:300) , Pax6 (Covance, PRB-278P, 1:400), cleaved-caspase 3 (Cell Signaling, 9661S, 1:500). The following day, sections were washed 3 times for 10 min each in PBS before incubation with secondary antibody (diluted 1:1000) and DAPI at room temperature for 2 h. Sections were washed a final time in PBS for 10 min 3 times before mounting in fluorescent mounting media (S3023, DAKO) .

### mRNA extraction, cDNA preparation and qPCR

mRNA was extracted from the cortices of E16.5 embryos (n = 5 for each line), snap-frozen using liquid nitrogen. For this, we followed the instructions of a commercially available kit (RNeasy Mini Kit, 74,104, Qiagen). Subsequently, the cDNA was synthesized using a different kit (QuantiTect Reverse Transcription Kit, Qiagen, 205,313). To quantify the levels of the most highly expressed α-tubulin genes in the mouse brain—*Tuba1a, Tuba1b, Tuba1c* and *Tuba4a*, we used SYBR green (SsoAdvanced Universal SYBR Green Supermix, 1,725,272, Biorad) on a Biorad CFX 384 Real Time Cycler. We used the following primers: mTuba1a_qPCR_IS_1F/TCTCTTACATCGACCGCCTAA and mTuba1a_qPCR_IS_1R/GCCAACATGGATGGAGATG, mTuba1b_qPCR_IS_1F/TCTCTCACCCTCGCCTTCTA and mTuba1b_qPCR_IS_1R/AGCTGCTCAGGATGGAAGAG, mTuba1c_qPCR_IS_1F/GCGGACCACTTCAAGGACTA and mTuba1c_qPCR_IS_1R/AGCTGCTCAGGATGGAAGAG, mTuba4a_qPCR_IS_1F/CGTACAGCCCAAACTCATCAT and mTuba4a_qPCR_IS_1R/AGAAGGTGGTGAAGGAGTCGT. In addition, we used three control genes: *Hprt* (Hprt_qPCR_F/GAACCAGGTTATGACCTAGATTTGTT and Hprt_qPCR_R/CAAGTCTTTCAGTCCTGTCCATAAT), *Tfrc* (Tfrc_qPCR_F/TCGCTTATATTGGGCAGACC and Tfrc_qPCR_R/ATCCAGCCTCACGAGGAGT) and *Pgk1* (Pgk1_qPCR_F/AAAGTCAGCCATGTGAGCACT and Pgk1_qPCR_R/ACTTAGGAGCACAGGAACCAAA). We compared three different genotypes and performed all reactions in technical triplicates. For each of the mouse lines, we ran all biological replicates (n = 5) together in one 384-well plate (1 plate per line). The 384-well plates were set up using an Agilent Bravo LT96 Liquid Handling system. We calculated the geometric mean of the Ct values for the three control genes and determined the ΔCt (difference to the mean of the Ct values for each tubulin gene assessed). The relative mRNA expression levels were calculated using the qPCR primer efficiency (between 95 and 105%) as previously described^33^.

### Statistical analysis

The statistical analysis was performed using the GraphPad Prism software (v8.0.2). To analyse our qPCR data, we employed a two-way repeated measures ANOVA, with a Tukey’s multiple comparisons test. We performed one-way ANOVA with Tukey’s multiple comparison test to analyse the number of Pax6 and Ctip2-positive cells between different genotypes. Quantitation of cleaved-caspase-3-postive cells was performed using Brown-Forsythe and Welch ANOVA tests and Dunnett's T3 multiple comparisons test. A summary of the statistical tests used in this study is included in Supplementary Material Table S1. All samples and animals used in this study were not subject to randomization but were assigned to experimental groups based on their genotype. All quantitation was performed blind to genotype.

## Supplementary Information

Supplementary Information 1.

Supplementary Information 2.

## Data Availability

The datasets generated and/or analysed during the current study are available from the corresponding author on reasonable request. The DNA sequences of the Tuba1a locus in our mice have been deposited on Genbank (BankIt2640849 Tuba1a OP819667, BankIt2642162 Tuba1a OP819668).
