# Supplementary material for: Codon modification of Tuba1a alters mRNA levels and causes a severe neurodevelopmental phenotype in mice
Source: Sci Rep. 2023 Jan 21;13:1215. doi: 10.1038/s41598-023-27782-2 (PMC9867703; doi:10.1038/s41598-023-27782-2)
Supplement: Supplementary file 1 — Supplementary Information 1. [file 41598_2023_27782_MOESM1_ESM.docx]

**Supplementary Information**

**Codon modification of *Tuba1a* alters mRNA levels and causes a severe neurodevelopmental phenotype in mice**

Ines Leca, Alexander William Phillips, Lyubov Ushakova, Thomas David Cushion, and David Anthony Keays

**b**

**a**

**
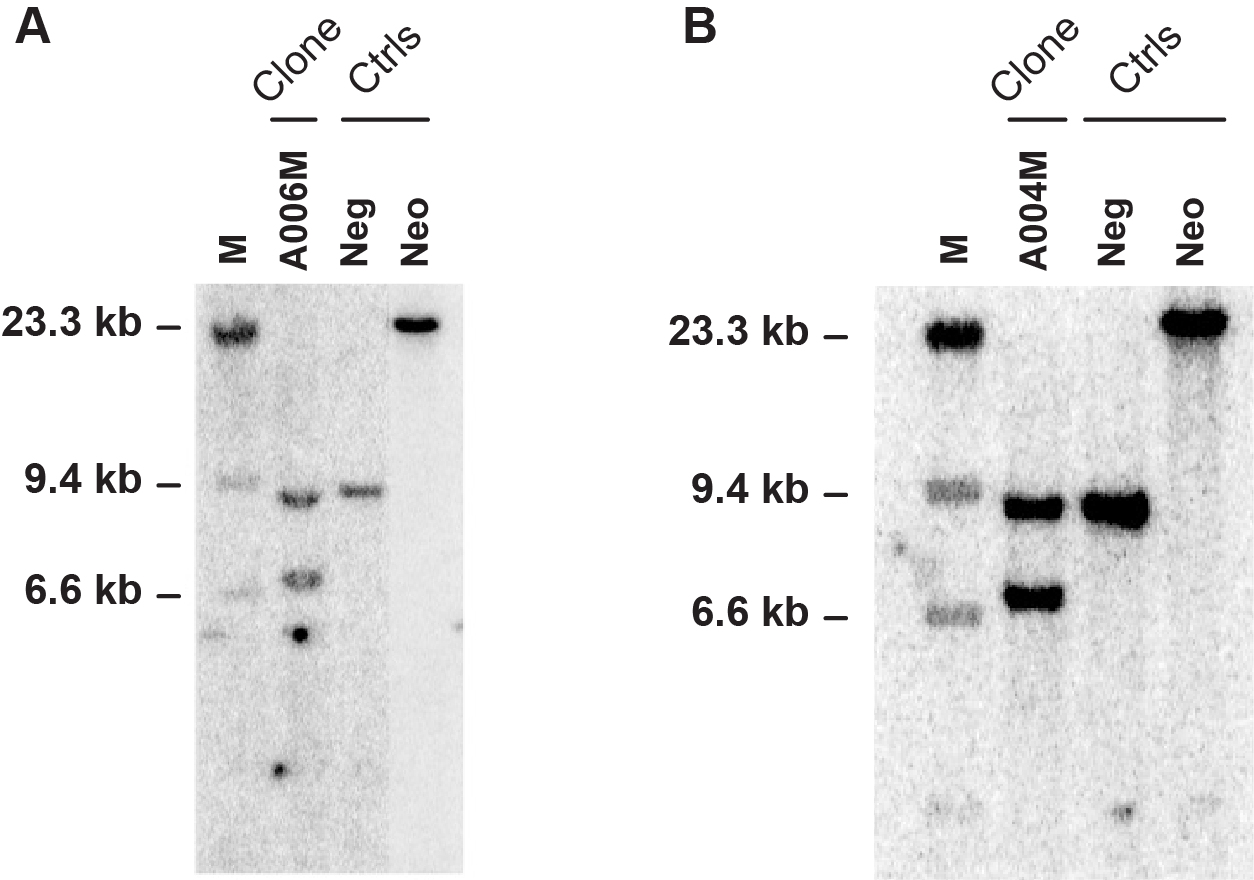

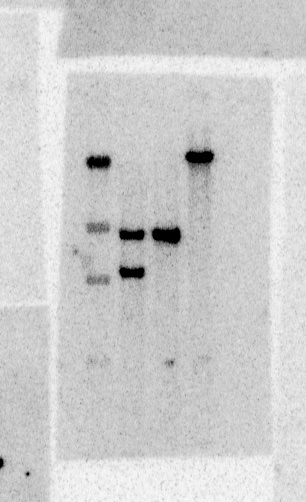
**

**Supplementary Figure 1 – Generation of *R402H Tuba1a (modified)* line, related to Figure 1.**

(**a**) Southern blot analysis of mice demonstrating successful targetting. Chimeric animals were backcrossed to C57/BL6. DNA from their offspring were digested with Nde_1, and probed with a 5’ southern probe. The expected sizes of the wild-type allele is 9.1kb and the conditional knock-in allele 7 kb. A004M is heterozygous for the *R402H Tuba1a (modified)* insert. Neg, shows a wild-type negative control. M indicates marker. (**b**) Shows the original uncropped version of the blot shown in (a).


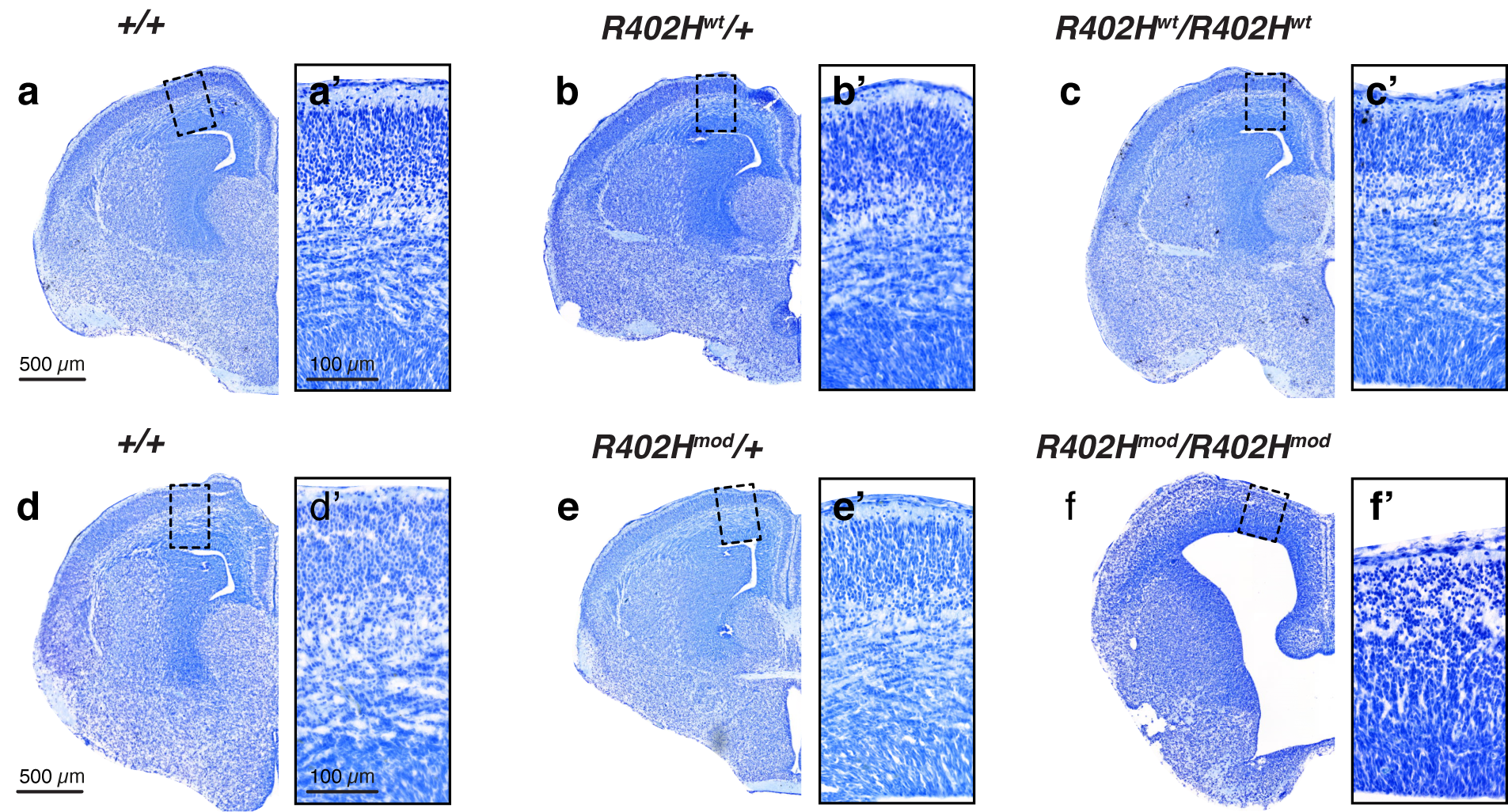


**Supplementary Figure 2 – Characterization of *R402H Tuba1a* *(wt)* and *R402H Tuba1a* *(modified)* lines, related to Figure 2.**

(**a-c**) Additional representative rostral coronal sections of E16.5 embryos stained with Nissl. Wild-type (+/+), heterozygous (*R402H^wt^/+*) and homozygous (*R402H^wt^/R402H^wt^)* animals show no differences in brain development. (**a’-c’**) Enlargements of the boxed areas marked in (**a-c**).

(**d-f**) Additional coronal sections of E16.5 embryos stained with Nissl of wild-type (+/+), heterozygous (*R402H^mod^/+*) and homozygous (*R402H^mod^/R402H^mod^*) animals. In rostral sections, *R402H^mod^/R402H^mod^* show a significant enlargement of the ventricles and a severe cortical disorganization. (**d’-f’**) Enlargements of the boxed areas marked in (**d-f**). Scale bars indicate 500 μm in (**a**) and (**d**), and 100 μm in (**a’**) and (**d’**).
